# Supplementary material for: Reversible conjugation of a CBASS nucleotide cyclase regulates bacterial immune response to phage infection
Source: Nat Microbiol. 2024 Apr 8;9(6):1579–92. doi: 10.1038/s41564-024-01670-5 (PMC11153139; doi:10.1038/s41564-024-01670-5)
Supplement: Supplementary file 1 — Supplementary Tables 1–6. [file 41564_2024_1670_MOESM1_ESM.pdf]

nature microbiology

Article

https://doi.org/10.1038/s41564-024-01670-5

Reversible conjugation of a CBASS nucleotide cyclase regulates bacterial immune response to phage infection

In the format provided by the
authors and unedited

Supplementary Table 1

Phages used in this study

Name	GenBank Accession number	Family	Subfamily	Genus	Group	Morphology	Lifestyle	Closest relative	
								**Name; accession**	**Identity; coverage**
vB_BsuM-Goe16	OM728298	*Herelleviridae*	*Spounavirinae*	*Okubovirus*	SPO1-like	Myovirus	lytic	*Bacillus* phage SP8; MW001214	53.3%; 24.84%
vB_BsuM-Goe21	OM728297	Unclassified	Unclassified	*Takahashivirus*	PBS-like	Myovirus	lytic	*Bacillus* phage PBS1; NC_043027	87.6%; 62.24%
vB_BsuP-Goe23	OM240929	*Salasmaviridae*	*Picovirinae*	*Salasvirus*	Phi29-like	Podovirus	lytic	*Bacillus* phage phi29; NC_011048	91.6%; 91.46%
vB_BsuM-Goe26	OM728296	*Herelleviridae*	*Spounavirinae*	Unclassified	SP10-like	Myovirus	lytic	*Bacillus* phage SP10; NC_019487	75.7%; 61.12%

The classifications of the phages correspond to their publication in NCBI under the respective GenBank accession number.

Supplementary Table 2

Bacillus subtilis strains used in this study

Name	Genotype	Construction	Reference
168	*trpC2*		[1]
Δ6	*trpC2; ΔSPß; sublancin 168-sensitive; Δskin ΔPBSX Δprophage1 pks::Cm Δprophage3*		[2]
LK06	*trpC2; ΔSPß; sublancin 168-sensitive; Δskin ΔPBSX Δprophage1 pks::Cm Δprophage3 lacA::(pdegQ cdntase+cap2+cap3+nuc-saved (Bce) aphA3)*	pLK01 → Δ6	This study
LK09	*trpC2; ΔSPß; sublancin 168-sensitive; Δskin ΔPBSX Δprophage1 pks::Cm Δprophage3 lacA::(pdegQ cdntase (Bce) aphA3)*	pLK04 → Δ6	This study
LK10	*trpC2; ΔSPß; sublancin 168-sensitive; Δskin ΔPBSX Δprophage1 pks::Cm Δprophage3 lacA::(pdegQ cdntase+nuc-saved (Bce) aphA3)*	pLK05 → Δ6	This study
LK18	*trpC2; ΔSPß; sublancin 168-sensitive; Δskin ΔPBSX Δprophage1 pks::Cm Δprophage3 lacA::(pdegQ aphA3)*	pGP1460 → Δ6	This study
LK19	*trpC2; ΔSPß; sublancin 168-sensitive; Δskin ΔPBSX Δprophage1 pks::Cm Δprophage3 lacA::(pdegQ cap2+cap3+nuc-saved (Bce) aphA3)*	pLK23 → Δ6	This study
LK20	*trpC2; ΔSPß; sublancin 168-sensitive; Δskin ΔPBSX Δprophage1 pks::Cm Δprophage3 lacA::(pdegQ cdntase+cap2+nuc-saved (Bce) aphA3)*	pLK29 → Δ6	This study
LK40	*trpC2; ΔSPß; sublancin 168-sensitive; Δskin ΔPBSX Δprophage1 pks::Cm Δprophage3 lacA::(pdegQ cap2+nuc-saved (Bce) aphA3)*	pLK43 → Δ6	This study
LK51	*trpC2; ΔSPß; sublancin 168-sensitive; Δskin ΔPBSX Δprophage1 pks::Cm Δprophage3 lacA::(pdegQ cdntase+cap2+cap3+nuc-saved (Bce) aphA3) ΔpspA::phleo*	LFH product → LK06	This study
LK63	*trpC2; ΔSPβ; sublancin 168-sensitive; Δskin ΔPBSX Δprophage1 pks::Cm Δprophage3 lacA::(pdegQwt cdntase+cap2+cap3+nucsaved (Bce) aphA3)*	pLK67 → Δ6	This study
LK64	*trpC2; ΔSPß; sublancin 168-sensitive; Δskin ΔPBSX Δprophage1 pks::Cm Δprophage3 lacA::(pdegQ cdntase+cap2-C91R+cap3+nuc-saved (Bce) aphA3)*	pLK64 → Δ6	This study
LK65	*trpC2; ΔSPß; sublancin 168-sensitive; Δskin ΔPBSX Δprophage1 pks::Cm Δprophage3 lacA::(pdegQ cdntase+cap2-C403R+cap3+nuc-saved (Bce) aphA3)*	pLK65 → Δ6	This study
LK66	*trpC2; ΔSPß; sublancin 168-sensitive; Δskin ΔPBSX Δprophage1 pks::Cm Δprophage3 lacA::(pdegQ cdntase-A331E+cap2+cap3+nuc-saved (Bce) aphA3)*	pLK68 → Δ6	This study
LK77	*trpC2; ΔSPβ; sublancin 168-sensitive; Δskin ΔPBSX Δprophage1 pks::Cm Δprophage3 lacA::(pdegQwt*	LFH → LK63	This study

	cdntase+cap2+cap3+nucsaved (Bce) aphA3)<br>*ΔpspA::phleo*	

Abbreviations: *Bsu*: *Bacillus subtilis*; *Bce*: *Bacillus cereus*; LFH: Long flanking homology PCR

Supplementary Table 3

Synthetic genes used in this study

Wild type CBASS operon *Bacillus cereus* WPySW2

ATGGCGACACAAAAGCAATTTTTAGCATTTTTAGGAGATATAGAGCCAAGTACAACAACTAAGGGTGATGCGAGTA
AGGCGCATACTGATTTAAGAAGTTTTTTGGAAAAGGATGCTACTTTTAAACCATACCGTGATTCAGATTTTTTATC
AGGATCATATAAACGGGATACAGCAATTCGTCCAAGAATTGTGGATGGAAAAATTACACGCCCCGATGTTGATATC
ATTGTAGTAACTAATTACACACAAGCAGATGACCCTAAAGATGTTATAAATTTACTGTATGATGTTTTAAAAAAAC
AATATCCTAATATCAGGAAACAAAATCGTTCAGTTGGAATAAATACAGGAAAGGCGGATATGGATGTTGTACCTAT
AATTGCACCTGATGGTATGGATGGAAAACTTTATATTCCTGATAGAAAACAAGAAAAGTGGCTTGAAACGAATCCC
CCTAAGCATACGATATGGACAATAGGGGTGAACCAAGAAAGCAAAGGTATGTTTAAACCACTTGTAAAAATAATGA
AGTGGTGGCGTCGTGTTAATCCAACAATAGCAAAGAAACCAAAAGGTTTTGTAATTGAGTGTATTGTAGCGGAATG
CATGGATAAATCTGAGACTAAATACGCTGAGCTATTCGTGAAAACTATGGAAGAAATTGTGAATAAATATGAGATC
TATGTTAGGTTAGGAATTGTCCCTACGATTGGTGATCCAGGTGTTCCAGGAAACTCTGTCACAGATGGAATTACAT
TTGATGCATTTAAAGGTTTTTACGATAAAGTCAAACTCCATGCCGAGATGGCGAGAAAAGCATTAGAAGAAACAAA
TGAAGATGAGGCTGTGAAATTGTGGCGTTCAATTTTCGGTCCGCGCTTCCCAAAGAGCAAAAAAAGTTCAAGTCCA
TTTGTGAAAAGTGCACTAATCCCACCACCGTTGACGTTTCCAGATCGTCCTATTGAACCAAAGAAACCCGGAGGAT
TTGCCTAATGGGGATTTGGTTCTTAGAAAATGCGAATCGTTTATTACATGAACGGAAAGAAATAGAACAATTACAA
TTAACTAAAGACTGGCTGCTAGGGATGGAATGGTGTATTTCAGGTAATGAACTAGCCGTACAAGTAAAAATAGAAG
CTCATGGTCATATTTATGAACTAGAAATGGGGTATCCACCTCTTTTCCCTTTCTCAGCACCAGGTGTTAAATCCAT
AGAGAAAGAGAAATGGTGGACGGAGCATCAGTATGTAAATGGAACTCTTTGTTTAGAATGGGGACCTGACAATTGG
CACGAAAATGTTACAGGTGCAAGAATCTTGGAAAGCGCCTACCGATTAATATATACGGAGAATCCAAAAGGAAAGC
AAGGTGGGGAAGAGAGTTCTGTTGTTCTTTCAAGACACGTACTCTCCGTTGGACAAGAACTAAGAACAGAAACGAT
TAGATTATATGTAAATCAGGTTCTGTTGGAAATAGTGTCAAATTCGAACCCTACTGAATATAAGCAAATGAATTTT
CACTACATTTTTGAAAATAGAGCTACCATTGTGCATATTGGACAGATTTATGATAAATCAAAAATAATTTGGGAAA
ACGATTATTTACCGAATGAATTGAAAGGTAAGCTATTAGGAAAAGGAAGCATTTATTATGTAAAGAATGCATCAGC
TGAAATTAAATCTCTATCTTCAAGTGAAGCATTAAAATATTTATTATTGAAAAACGGATATAAAGAGGAATGTATT
GAATTTGAAGATTTAACCAATAGTTTATTTATATTGGTTGATGAGGTAGGAGCGATATTTTCGCTATTATGTATTA
AAAATAAAGAAAAGATACATAAGATACCAATAGTAATGGACAAAGAAAACGTGGATCGGCGCCAACCGGAATTGTT
TTCACTAAAAGATAAAAAGGTAGGTATGGTTGGAGTAGGCTCTTTAGGAAGCAAAATAGTGAATGCTCTTGCAAGA
TCAGGGGTAGAGAAATTTTTCCTTGTTGATGAAGATATATTTTTGAGAGGGAATATTCAGAGGCATACCTTGGATT
GGAGAAATGTCGGGAATCATAAGGTTGATGCTATTAAAGAGCAGCTTGAACTTATTTCAAGCTCTGTAGAAGTTGA
AGTATCTAGGATCAACTTAACTGGGCAAGAAGCAATTACATCATTAAATGCGAATATTACTGAATTAGGCTCTTGT
GACGTTATTGTTGATGCTACGGCGGATAGTAAGATTTTTAATTTGTTATCTGCGATATGTAAAAATTATGAGAAAC
CAATGATTTGGGGAGAATTATTTTCTGGTGGGATCGGGGGGCTTATTGCAAGAAGTCGTCCCAAGTTAGATCCAAG
TCCTCAAATTATGAGAAAGGCACTTTTGGAAGCTACTTCAGAAATGCCTACAATATCTCAATTAGGCGAAGAGGCG
TATGGATTACAAACTGAATTAGGTGAAGTATGGATTGCTTCAGATGCTGATGTAGGCATTATTGCGAATCATTTAG
CAAGGTATATAACTGATGCACTTAAAGATGAAAAGTCGGATTATCCTTTTTCTATGTATTTAATAGGTTTGAAGAA
AAGCTGGATATTTGAACAACCTTTTGAAATATTTCCGATTGAGACGAATCATTTAATTGAGGAAATAATACCGGAG
GAAAATCAAGATGTTATAAAAGAAGTAATTGATTTTATAGCGCCGTTATTGGAGAAGAGAAATGATTAAAGTTATT
ATGCCTAAAAGAATTGAACAAAGATTGGAAGTTGAGCTTAAAAAGGCAGGCAGAAATGAAATTGGTGGCATTTTAA
TGGGAGAGCATATCGGAGAAAATACATTTAGAATTTGTGATGTGACGGTCCAATTATATGATGGTACATGGATTCG
TTTCGTAAGACAAATATCAGGTGTAATGAGAAAAAGTCTAAATTACTTTTTTAGTAAAAATAAGTTCCAATATAGA
AAATATAATTATCTAGGTGAGTGGCACTCACATCCTTCTTTTTCGCTTATGCCTAGTTCTCAAGATATTCAAACGA
TGTGGGGAATAGTAAATGATGAAGCTGTAGGTGCAAATTTTGCTGTTTTGTTAATTGTAAAAATAGAATCTCAATC
TGTACATGGCAATGTTACGCTTTACGTACCTGGTAACCCTGTATTGAAAGGTGAGATGGTAAAGGGAGGGGATTAT
ACATGAATGAACACAAGCCATCTTTATTGGAATCGGAATCGACTGGGGGAGACATTGCAGGGGGAGGATTTGATTT
CCAAAGAAATTTAATCCTCAATAAAATTCCTTATTGGCTTTCTTTCGAAGGGTTTACATCCCTTATTTGGGAATCG
ATTGGGGATATTGAAGTGAAGTTTTTTGTTCCAGGCAAAGGTATGATTATTGAAGCGATTGAGGCTAAGAATCATA
ATATGACGCCAGCAAAATTTTGGGAAGAGATCGAACGATTTAAAACAATGGATAAGGGGAGTCCAGGTACATATCG
CTGGTTCACTTTATCTTGTACAGGGGTATCAGATACAATTAAACCCTTAATTAACGGATTACGAAGACTGAGAGAT
CCATATTCTTTTTTTGAACAATCATCAGGGATTCTACAGAATTCATATGAAGCTTATAAACAAATTGTTTTGAAAC
TTGAAAAAGATGAGGAAACAGCAGAGTTTTTGTTTAATAAGGTTATGATTGAAGATACATGGGGATCATTGAATGC
ACAGTCGGAAGGGATGTTTTTTGGGAATTTGTCAGAAAACCTTCCAGATTTTGATGAACTACCTAAGAAGAAAATA
AATAATGTCCATAGTTATTTAACAGAGTTGCTGGTATCAAGAAAAAATAAGCCAGTTTCAAGAAAAGAAATTAAAG

AAACAATTATACGTTCTATTGAAGATGATGAATTTTTTTCAAAACCAACGATTCTTGAGACGAAAATAAATAATGA
GGAAACAAGTGGAAAACAATTGTTGTTTATGTGGGAACCGTTTTTCGGAGGAAAGGAAAGAAGCTTTCCTTCATCT
GAAGAGTGGACTAGCCAATTGCTTACTGAATTAGAGCAAACAAAACAGTGGATTATCGATAATAGGACTAATCGTA
AAATCAGATTGCAAGGAAGTAGAAGGAATTCAAGCGCGATGGCAATAGGTCAAACCTTTTCTGCTGTTTCGGGATT
TAATATTGAAATGGAGTATAGAGGAGATTTCTGGAGTACAAATCAATACCCTACATCAACCACACCAGGGTATCCA
ATTCAAACTAACTTCCGGGTAGGAAAAGGAAAAAGCTTGCTGTGATAATTGCAATAATGAAAGAAAATATGACAG
ATGAGGTTAGAACGTTTTTAAGCGGAATTGATGAGGAAGAAAATTCGGTATTAGAGGTAGCATCATCATTTCCAAT
CGTTTCGGCTGAACAAGTAAATGTAGTTGTAAACGCAATAAAAGAAGAAATAAAGAAAGTATGTGCACAAATTGAT
GTAGACGAAATTGATTTGTTTTATGCTGGACCATCTCATCTTGCTTTATTCTTAGGACATTGCTGGAATGCGATGC
CTCGTACTCAATGTTATGAATGGGTTAAAACTGGAGATTATGTAAAAACAGTTTGTTTATCTTGA

Codon optimized sequence of CBASS genes

QJU33211.1 nucleotidyltransferase [Bacillus cereus WPySW2]

ATGGCAACCCAGAAACAATTTCTGGCCTTTTTAGGAGATATTGAACCATCGACAACTACGAAAGGGGATGCCAGCA
AAGCCCACACCGATTTACGTTCATTTTTGGAGAAAGACGCTACATTTAAGCCATATCGCGATTCAGATTTCCTTAG
CGGCTCGTACAAGCGCGATACAGCGATCCGCCCTCGTATTGTTGACGGCAAAATCACTCGTCCAGATGTCGATATT
ATTGTGGTGACAAATTATACCCAGGCTGATGATCCAAAGGACGTTATTAACCTTTTATACGATGTTTTAAAAAAAC
AGTATCCCAACATTCGTAAACAAAATCGTTCAGTCGGTATTAACACCGGAAAAGCAGATATGGATGTGGTGCCTAT
TATTGCCCCGGATGGGATGGACGGAAAACTTTATATTCCAGATCGTAAGCAGGAGAAATGGCTGGAGACGAATCCT
CCCAAGCATACTATCTGGACAATCGGTGTCAACCAAGAGAGCAAGGGGATGTTCAAACCCTTGGTAAAAATTATGA
AATGGTGGCGTCGCGTCAATCCTACTATCGCAAAGAAGCCTAAGGGTTTTGTTATCGAGTGCATCGTCGCCGAGTG
CATGGATAAATCCGAAACTAAGTACGCTGAGCTTTTTGTGAAAACTATGGAGGAAATTGTGAATAAATACGAAATT
TACGTCCGTTTAGGTATCGTACCCACCATTGGGGACCCCCAGGAGTGCCCGGCAATTCCGTCACTGATGGTATCAC
CTTCGACGCCTTCAAAGGGTTTTACGATAAGGTAAAATTACATGCTGAAATGGCGCGTAAAGCTCTGGAAGAGACC
AATGAGGACGAGGCAGTTAAACTGTGGCGTTCGATCTTTGGGCCACGTTTCCCTAAATCGAAGAAGTCGTCCAGTC
CTTTTGTAAAATCGGCACTGATTCCTCCGCCGTTGACATTCCCGGACCGTCCGATCGAGCCAAAAAAACCCGGAGG
TTTCGCCTGA

QJU33212.1 hypothetical protein HLB41_05150 [Bacillus cereus WPySW2]

ATGGGAATTTGGTTTCTTGAGAATGCTAACCGCCTTTTACACGAACGCAAGGAGATTGAACAACTTCAACTTACGA
AGGACTGGTTATTGGGGATGGAATGGTGTATTAGTGGCAATGAACTTGCAGTTCAGGTCAAAATCGAGGCGCACGG
GCATATCTATGAGCTGGAGATGGGCTATCCGCCTTTGTTTCCGTTTAGTGCGCCGGGGGTCAAGTCTATTGAAAAG
GAGAAGTGGTGGACAGAACATCAGTACGTAAACGGCACCTTGTGTCTTGAGTGGGGCCCTGATAACTGGCACGAGA
ATGTCACGGGTGCGCGCATTCTTGAATCTGCTTATCGCTTAATCTATACCGAAAACCCCAAAGGGAAACAGGGAGG
GGAAGAATCCAGTGTTGTGTTGTCACGCCACGTGCTGTCTGTTGGGCAGGAACTTCGTACTGAGACAATTCGCCTG
TACGTCAATCAGGTACTGCTTGAGATTGTGTCGAATAGTAACCCCACTGAGTATAAACAGATGAATTTCCACTACA
TCTTCGAGAACCGTGCCACCATCGTCCACATTGGGCAGATTTACGATAAGTCGAAAATCATCTGGGAAAATGACTA
TTTGCCCAACGAACTTAAGGGGAAACTGTTAGGTAAGGGTTCCATTTATTATGTCAAAAATGCCAGCGCAGAGATT
AAATCATTATCATCGTCGGAGGCGTTGAAGTATTTACTGTTGAAAAACGGGTATAAAGAGGAATGTATTGAATTTG
AGGACTTAACCAATTCTCTGTTTATCTTGGTTGATGAAGTAGGCGCAATTTTCTCGCTGCTTTGTATCAAGAATAA
GGAGAAGATTCATAAGATTCCGATTGTCATGGATAAAGAGAATGTGGACCGCCGCCAGCCCGAGTTATTTAGCTTG
AAAGATAAAAAAGTCGGGATGGTTGGTGTAGGAAGCTTGGGGTCAAAAATTGTGAATGCCTTAGCGCGCAGCGGGG
TCGAGAAGTTCTTCTTGGTTGATGAGGACATCTTCTTGCGCGGAAATATCCAGCGCCATACATTGGATTGGCGTAA
TGTGGGTAATCATAAGGTCGATGCCATTAAAGAACAATTGGAACTGATCTCGTCATCAGTTGAAGTGGAAGTTTCT
CGTATTAATCTGACAGGACAGGAAGCGATTACCTCACTGAATGCGAATATTACAGAATTAGGCTCTTGTGATGTGA
TTGTCGATGCGACGGCGGATTCGAAGATTTTTAACTTACTGAGTGCCATTTGCAAGAATTATGAAAAGCCTATGAT
TTGGGGGGAATTGTTTAGTGGAGGGATCGGGGGGCTGATCGCCCGCTCGCGCCCAAAGTTAGACCCATCTCCGCAA
ATTATGCGCAAGGCCCTTCTTGAGGCTACATCGGAAATGCCCACAATCTCACAATTGGGTGAAGAGGCATACGGCT
TGCAGACGGAATTGGGAGAAGTATGGATCGCATCGGATGCCGACGTGGGAATCATTGCTAATCATTTGGCGCGCTA
TATCACCGACGCTTTGAAAGATGAGAAGTCTGACTATCCTTTTTCGATGTATCTTATTGGACTGAAAAAGTCGTGG
ATCTTTGAACAGCCGTTCGAAATCTTTCCAATCGAAACCAACCATCTGATCGAAGAAATTATTCCTGAAGAGAATC
AGGACGTGATCAAAGAGGTGATCGATTTCATTGCCCCTTTGCTGGAAAAACGCAACGATTGA

WP_217635926.1 Mov34/MPN/PAD-1 family protein [Cytobacillus oceanisediminis]

ATGGCACATATGATTAAAGTCATTCTTCCGAAGTCCATCGAAAACAAAATGCTTGCCGAGCTGAAGAAAGCAGGTT
CACGTGAGATCGGAGGAGTGCTGATGGGGGAACATATCGAGGAGAATATCTATAAGATTTATGACATCACGGTCCA
GTCCCAAGGGGGATCATGGATTAGTTTCGTTCGCCATTTAAGTATTTCTATGAAAAGCGCATTAAACCGTTTCTTT
CAAAAAACTAATTACGAGTATTCCAAATACAACTATTTGGGTGAGTGGCATAGCCATCCCTCGTTCAACTTGTCTC
CTTCAAGCCGCGACATCCAGACCATGATCGAAATCGTCAATGATAAGAACGTAGGCGCGAATTTCGCGATTTTACT

GATCGTTAAGTTTGAGAATGAACTGGTTGGAGATATCACATTGTTTGTGCCAAATTACCCAATCCTGAAGGGTGAG
CTTTTTCGCGAAGAGGAAGACGTACACGAGTGA

QJU33214.1 SAVED domain-containing protein [Bacillus cereus WPySW2]

ATGAACGAGCACAAACCGTCTCTGTTGGAAAGCGAAAGTACGGGTGGTGACATCGCCGGGGGAGGATTCGATTTTC
AACGTAATTTGATCTTAAATAAGATCCCGTATTGGCTTTCTTTCGAAGGGTTCACTTCGCTTATCTGGGAGTCCAT
CGGAGATATCGAAGTAAAGTTTTTCGTCCCTGGGAAGGGGATGATTATCGAGGCCATTGAGGCAAAAAACCACAAT
ATGACCCCGGCGAAATTCTGGGAGGAGATCGAGCGCTTTAAAACTATGGATAAAGGATCGCCTGGGACTTATCGCT
GGTTCACGCTGTCGTGCACTGGCGTCTCAGACACCATCAAGCCACTTATCAACGGCTTACGCCGTCTGCGTGACCC
ATACTCTTTTTTTGAGCAGAGCTCTGGCATTCTTCAGAATTCTTATGAGGCATACAAACAGATTGTCCTGAAATTA
GAGAAGGACGAGGAAACGGCAGAGTTCTTGTTCAATAAAGTCATGATTGAGGACACGTGGGGTAGTTTGAACGCAC
AATCGGAGGGTATGTTTTTCGGGAATCTTTCCGAGAATTTGCCCGATTTCGACGAGTTACCTAAAAAAAAGATCAA
CAACGTACACTCCTATCTTACCGAATTATTAGTATCGCGCAAAAACAAGCCTGTATCGCGTAAGGAGATTAAGGAG
ACTATCATTCGCAGTATCGAGGATGACGAATTTTTTTCCAAGCCCACGATCTTAGAGACGAAGATCAATAACGAGG
AAACGTCTGGTAAACAGTTACTTTTTATGTGGGAACCGTTCTTTGGCGGGAAAGAACGTTCGTTTCCGTCGTCGGA
AGAGTGGACCAGCCAGCTGCTTACTGAATTGGAACAGACGAAGCAATGGATCATTGACAACCGTACGAACCGCAAG
ATTCGCTTACAGGGAAGCCGCCGCAACTCTTCAGCTATGGCCATTGGTCAGACGTTCTCCGCTGTAAGTGGTTTCA
ATATTGAGATGGAGTATCGTGGAGACTTCTGGTCAACCAATCAATACCCCACGAGCACCACTCCTGGGTATCCCAT
TCAGACTAATTTCCGCGTAGGCAAGGGGAAAAAGCTGGCCGTTATCATTGCAATTATGAAGGAAAATATGACCGAC
GAGGTCCGCACGTTTCTGTCGGGCATCGATGAGGAAGAGAATTCCGTTTTGGAGGTTGCTTCTAGCTTCCCTATTG
TATCGGCTGAACAAGTCAATGTTGTCGTCAACGCGATTAAAGAGGAGATTAAGAAAGTGTGCGCTCAAATCGACGT
AGACGAGATTGATTTATTTTACGCGGGACCCTCACATCTTGCATTATTCCTGGGGCACTGCTGGAACGCCATGCCA
CGCACCCAGTGTTACGAATGGGTTAAAACAGGGGACTACGTGAAAACCGTGTGTCTGAGCTGA

QJU32920.1 PspA/IM30 family protein [Bacillus cereus]

GCGCCCATGGCACATATGTCCGTGTTCAAACGCCTGCGCGACCTGACAATGAGTAACGTATATAGCTTGATCGAGA
AGGCCGAGGACCCAGTTAAGATGACTGATCAATACTTGCGTGACATGCAAGCGGACGTGCAAGAAGCGGAGAAATC
TGTGGCCGCTCAAATTGCACTGGAGAAAAAATTTAAAATTCTTTTCGAGGAGCAAGAAGCCCTGGTAAAGAAACGT
GAGGAGCAAGCTCACATGGCCGTTCAGGCCAACAATCTTGACCTGGCCCGTCGTGCGTTGGAAGAAAAACAAAACG
CCGAACAGAAAATGAACGAGTACAAGGCATCTTACGAGCAGAACAAGGCGGCTGCCGATAATCTTCGCTTAAAGCT
GGAAGAAATGCGTAAGCAACTGACTGAACTGAAAAACAAACGCGAAACACTGGTGGCCCGTGTGAACGCGGCTAAA
GCGCAAAAAAACATTAATCAAGCCATGTCTGGTTTTGATTCCAATTCAGCCAAAGCGGGACTGAGTCGCATGGAAG
AGAAAGCGCTTCAGCTGGAAGCAGAAGCCGAAGCAAGCGGGGAAGTCTACAAAAAGGAGAAGTCTCTGGATGATGA
GTTCGCGAGCCTGAATAAAAACTCAGCCGTCGATGATGAATTGGCGCGTATCATGAAGCAATATGAAAAGTGACTC
GAGGGATCCCGCG

Supplementary Table 4

Plasmids used in this study

Name	Vector	Insert	Reference
pGP172		-	[3]
pGP380		-	[4]
pGP1460		-	[5]
pLK01	pGP1460/BamHI+XbaI	*cdntase+cap2+cap3+nuc-saved* (*Bce*) (KL01+KL02)	This study
pLK04	pGP1460/BamHI+XbaI	*cdntase* (*Bce*) (KL01+KL05)	This study
pLK05	pLK04/XbaI+SalI	*nuc-saved* (*Bce*) (KL09+KL10)	This study
pLK20	pGP380/BamHI+SalI	*cdntase* (*Bce*) (KL28+KL29)	This study
pLK21	pGP380/BamHI+SalI	*cdntase* (*Bce*) (truncation after K300) (KL28+KL30)	This study
pLK23	pGP1460/BamHI+XbaI	*cap2+cap3+nuc-saved* (*Bce*) (KL43+KL02)	This study
pLK29	pLK03/XbaI+SalI	*nuc-saved* (*Bce*) (KL09+KL10)	This study
pLK37	pGP172/SacI+BamHI	Cap2 (*Bce*) (KL73+KL79)	This study
pLK38	pLK33/BamHI+EcoRI	Cap3 (*Bce*) (KL75+KL76)	This study
pLK43	pGP1460/BamHI+SalI	Cap2+Nuc-SAVED (*Bce*) (KL43+KL10)	This study
pLK44	pEhisV5tev/PspA	*pspA* (*Bsu*) (KL113+KL114)	This study
pLK54	pLK44	PspA-K220R (*Bsu*) (KL115+KL116)	This study
pLK56	pLK48/XbaI+SalI	*nuc-saved* (*Bce*) (KL09+KL10)	This study
pLK61	pLK44	PspA-K147R (*Bsu*) (KL133+KL134)	This study
pLK64	pLK01	C91R in Cap2 *(Bce)* (KL135+136)	This study
pLK65	pLK01	C403R in Cap2 *(Bce)* (KL137+138)	This study
pLK67	pLK01	PdegQ36 promoter -10 T→C degQwt (KL139+KL140)	This study
pLK68	pLK01	A331E CD-NTase (*Bce*) (KL144+145)	This study
pLK69	pLK01	A331G CD-NTase (*Bce*) (KL146+147)	This study
pEhisV5TEV/ *cd-ntase*	pEhisV5TEV	*cdntase* (*Bce*)	This study
pEhisV5TEV/ *cd-ntaseΔC*	pEhisV5TEV	*cdntase ΔC* (*Bce*), aa1-301	This study
pEhisV5TEV/ *nuc-saved*	pEhisV5TEV	*nuc-saved* (*Bce*)	This study
pEhisV5TEV/ *cap2*	pEhisV5TEV	*cap2* (*Bce)*	This study
pEhisV5TEV/ *cap2C91R*	pEhisV5TEV/ *cap2*	C91R in *cap2 (Bce)*	This study
pEhisV5TEV/ *cap2C403R*	pEhisV5TEV/ *cap2*	C403R in *cap2 (Bce)*	This study
pEhisV5TEV/ *cap3*	pEhisV5TEV	*cap2* (*Coc)*	This study

Abbreviations: *Bsu*: *Bacillus subtilis*; *Bce*: *Bacillus cereus; Coc: Cytobacillus oceanisediminis*

Supplementary Table 5

Oligonucleotides used in this study

Name	Sequence	Gene	Comments
KL01	AAAGGATCC**ATTAAAGAGGAGAAATTAACT**ATGGCGACACAAAAGCAATTTTTAG	fw, *cdntase* (*Bce*)	BamHI, +RBS
KL02	TTTTCTAGATCAAGATAAACAAACTGTTTTTACATAATCTC	rev, *nuc*-saved (*Bce*)	XbaI, with STOP
KL05	TTTTCTAGATTAGGCAAATCCTCCGGGTTTC	rev, *cdntase* (*Bce*)	XbaI, with STOP
KL09	AAATCTAGA**ATTAAAGAGGAGAAATTAACTATGA**ATGAACACAAGCCATCTTTATTG	fw, *nuc*-saved (*Bce*)	XbaI, +RBS
KL10	TTTGTCGACTCAAGATAAACAAACTGTTTTTACATAATCTC	rev, *nuc*-saved (*Bce*)	SalI
KL28	AAAGGATCCATGGCGACACAAAAGCAATTTTTAG	fw, *cdntase* (*Bce*) into pGP380	BamHI
KL29	TTTGTCGACTTAGGCAAATCCTCCGGGTTTC	rev, *cdntase* (*Bce*) into pGP380	SalI
KL30	TTTGTCGAC**TTA**TTTTTTGCTCTTTGGGAAGCGC	rev, *cdntase* (truncation after K300) (*Bce*) into pGP380	SalI, + STOP
KL43	AAAGGATCC**ATTAAAGAGGAGAAATTAACT**ATGGGGATTTGGTTCTTAGAAAATGC	fw, *cap2* (*Bce*)	BamHI, +RBS
KL73	AAAGAGCTC**A**GGGATTTGGTTCTTAGAAAATGCGA	fw, *cap2* (*Bce*) into pGP172	SacI; one extra base to restore reading frame
KL79	TTTGGATCCTTAATCATTTCTCTTCTCCAATAACGG	rev, *cap2* (*Bce*) into pGP172	BamHI
KL113	AAACCATGG**A**ATGAGTATAATTGGAAGATTTAAAGATATTATGTC	fw, *pspA* (*Bsu*)	NcoI; one additional base to restore reading frame
KL114	AAACCATGG**AG**ATGAGTATAATTGGAAGATTTAAAGATATTATGTC	fw, *pspA* (*Bsu*) into pEhisV5tev	NcoI; two additional bases to restore reading frame
KL115	GCGGCGTTA**CGT**GCGAAAATGA	mutagenesis primer 1 K220R PspA (*Bsu*)	
KL116	CATCATTTTCGC**ACG**TAACGCC	mutagenesis primer 2 K220R PspA (*Bsu*)	
KL133	GCAGTAGCGCGTACGCAAGAAAG	mutagenesis primer 5 K147R PspA (*Bsu*)	
KL134	CATTCTTTCTTGCGTACGCGCTAC	mutagenesis primer 6 K147R PspA (*Bsu*)	
KL135	GGAACTCTT**C**GTTTAGAATGGGG	mutagenesis primer 1 C91R Cap2 (*Bce*)	
KL136	GGTCCCCATTCTAAAC**G**AAGAG	mutagenesis primer 2 C91R Cap2 (*Bce*)	

KL137	GAATTAGGCTCT**C**GTGACGTTATTG	mutagenesis primer 3<br>C403R Cap2 (*Bce*)
KL138	CATCAACAATAACGTCAC**G**AGAGCC	mutagenesis primer 4<br>C403R Cap2 (*Bce*)
KL139	CCGAAAGCAGACACACTATTAGTAAC	mutagenesis primer 1<br>PdegQ promoter -10<br>T->C degQwt
KL140	GATCTGTTACTAATAGTGTGTCTGCTTTC	mutagenesis primer 2<br>PdegQ promoter -10<br>T->C degQwt
KL144	GGAGGATTTGAGTAATGGGGATT	mutagenesis primer 1<br>A331E CD-NTase (*Bce*)
KL145	CCAAATCCCCATTACTCAAATCC	mutagenesis primer 2<br>A331E CD-NTase (*Bce*)
ΔC F	GCGCCCATGGCACATGAGCCCAAATTCCA ACCCCT	mutagenesis primer 1<br>aa1-301 *cdntaseΔC (Bce)*
ΔC R	CGCGGGATCCCTCGAGTCACGCCACCTTG CTGTCGTC	mutagenesis primer 2<br>aa1-301 *cdntaseΔC (Bce)*

Underlined: restriction sites; bold: additional bases/mutation

Abbreviations: RBS: ribosomal binding site; *Bsu*: *Bacillus subtilis*; *Bce*: *Bacillus cereus*

Supplementary Table 6: modified peptides identified by MS

Protein	Mass (kDa)	#PSMs	#Peptides
	**Ext. Data Fig. 2a Band 1 (KL18)**		
cyclase	38	117	22
$PspA^{Bsu}$	25	96	15
Cap2	68	7	7
	**Ext. Data Fig. 2b Band 2 (KL35)**		
$YdjI^{Bsu}$	36	143	17
	**Ext. Data Fig. 2b Band 3 (KL38)**		
cyclase	38	93	20
$PspA^{Bsu}$	25	83	22
	**Ext. Data Fig. 2b Band 4 (KL41)**		
$PspA^{Bsu}$	25	74	23
	**Ext. Data Fig. 4a Band 1 (KL44)**		
cyclase	37.52	18	12
Strep-Cap2	68	29	16
	**Ext. Data Fig. 4a Band 2 (KL45)**		
cyclase	37.52	55	21
Strep-Cap2	68	73	22
	**Ext. Data Fig. 4a Band 3 (KL46)**		
cyclase	37.52	62	26
Strep-Cap2	68	106	24
	**Ext. Data Fig. 4a Band 4 (KL47)**		
cyclase	37.52	89	24
Strep-Cap2	68	88	25
$PspA^{Bsu}$	25.2	44	17
	**Ext. Data Fig. 4c Band 4 (KL80)**		
cyclase	37.52	67	19
Strep-Cap2	68	581	50
$PspA^{Bce}$	25.2	72	26
	**Ext. Data Fig. 9a Band 1**		
cyclase	38	263	29
$PspA^{Bsu}$	25	64	15
	**Ext. Data Fig. 9a Band 2**		
cyclase	38	345	27
$PspA^{Bsu}$	25	111	18
	**Ext. Data Fig. 9a Band 3**		
cyclase	38	261	28
$PspA^{Bsu}$	25	113	21

References

1. Spizizen J. Transformation of biochemically deficient strains of *Bacillus subtilis* by deoxyribonucleate. *P Natl Acad Sci USA* **44**, 1072–8 (1958).

2. Westers, H. *et al.* Genome Engineering Reveals Large Dispensable Regions in *Bacillus subtilis*. *Mol. Biol. Evol.* **20**, 2076–2090 (2003).

3. Merzbacher, M., Detsch, C., Hillen, W. & Stülke, J. *Mycoplasma pneumoniae* HPr kinase/phosphorylase: Assigning functional roles to the P-loop and the HPr kinase/phosphorylase signature sequence motif. *Eur. J. Biochem.* **271**, 367–374 (2004).

4. Herzberg, C. *et al.* SPINE: A method for the rapid detection and analysis of protein-protein interactions *in vivo*. *Proteomics* (2007) doi:10.1002/pmic.200700491.

5. Mehne, F. M. P. *et al.* Cyclic di-AMP homeostasis in *Bacillus subtilis*. *J. Biol. Chem.* **288**, 2004–2017 (2013).
