# Supplementary material for: Reversible conjugation of a CBASS nucleotide cyclase regulates bacterial immune response to phage infection
Source: Nat Microbiol. 2024 Apr 8;9(6):1579–92. doi: 10.1038/s41564-024-01670-5 (PMC11153139; doi:10.1038/s41564-024-01670-5)
Supplement: Supplementary file 2 — Reporting Summary [file 41564_2024_1670_MOESM2_ESM.pdf]

nature portfolio

Corresponding author(s): Malcolm F White
Larissa Krüger

Last updated by author(s): Mar 5, 2024

Reporting Summary

Nature Portfolio wishes to improve the reproducibility of the work that we publish. This form provides structure for consistency and transparency in reporting. For further information on Nature Portfolio policies, see our Editorial Policies and the Editorial Policy Checklist.

Statistics

For all statistical analyses, confirm that the following items are present in the figure legend, table legend, main text, or Methods section.

n/a	Confirmed	
☐	☒	The exact sample size (*n*) for each experimental group/condition, given as a discrete number and unit of measurement
☐	☒	A statement on whether measurements were taken from distinct samples or whether the same sample was measured repeatedly
☐	☒	The statistical test(s) used AND whether they are one- or two-sided<br>*Only common tests should be described solely by name; describe more complex techniques in the Methods section.*
☒	☐	A description of all covariates tested
☒	☐	A description of any assumptions or corrections, such as tests of normality and adjustment for multiple comparisons
☐	☒	A full description of the statistical parameters including central tendency (e.g. means) or other basic estimates (e.g. regression coefficient) AND variation (e.g. standard deviation) or associated estimates of uncertainty (e.g. confidence intervals)
☐	☒	For null hypothesis testing, the test statistic (e.g. *F*, *t*, *r*) with confidence intervals, effect sizes, degrees of freedom and *P* value noted<br>*Give P values as exact values whenever suitable.*
☒	☐	For Bayesian analysis, information on the choice of priors and Markov chain Monte Carlo settings
☒	☐	For hierarchical and complex designs, identification of the appropriate level for tests and full reporting of outcomes
☒	☐	Estimates of effect sizes (e.g. Cohen's *d*, Pearson's *r*), indicating how they were calculated

Our web collection on statistics for biologists contains articles on many of the points above.

Software and code

Policy information about availability of computer code

Data collection	Phosphorimage and fluorescence scan data were collected using GE Healthcare ImageQuant TL 7.0<br>HPLC data were collected using Chromeleon 6.8 Chromatography Data System software (ThermoFisher). Western blots were visualized using the Licor Odyssey CLx Image Studio 5.x software.
Data analysis	GraphPad Prism 9.5.1 was used for enzymatic data analysis and creation of graphs. Geneious Prime 2022.2.1 (Biomatters) was used for sequence analysis. ImageJ 1.53k was used for quantification of gel images

For manuscripts utilizing custom algorithms or software that are central to the research but not yet described in published literature, software must be made available to editors and reviewers. We strongly encourage code deposition in a community repository (e.g. GitHub). See the Nature Portfolio guidelines for submitting code & software for further information.

Data

Policy information about availability of data

All manuscripts must include a data availability statement. This statement should provide the following information, where applicable:

- Accession codes, unique identifiers, or web links for publicly available datasets
- A description of any restrictions on data availability
- For clinical datasets or third party data, please ensure that the statement adheres to our policy

Supplementary information is available for this paper. Raw data are provided in the supplementary tables and files. Mass spectrometry data are available on

Figshare (dx.doi.org/10.6084/m9.figshare.25341769). The Bacillus subtilis complete proteome dataset UniProt Proteome ID UP000001570 is available at https://www.uniprot.org/. Correspondence and requests for materials should be addressed to Malcolm F White (mfw2@st-andrews.ac.uk) or Larissa Krüger (lmak1@st-andrews.ac.uk). For the purpose of open access, the author has applied a CC BY public copyright Licence to any Accepted Author Manuscript version arising from this submission.

Research involving human participants, their data, or biological material

Policy information about studies with human participants or human data. See also policy information about sex, gender (identity/presentation), and sexual orientation and race, ethnicity and racism.

Reporting on sex and gender	n/a
Reporting on race, ethnicity, or other socially relevant groupings	n/a
Population characteristics	n/a
Recruitment	n/a
Ethics oversight	n/a

Note that full information on the approval of the study protocol must also be provided in the manuscript.

Field-specific reporting

Please select the one below that is the best fit for your research. If you are not sure, read the appropriate sections before making your selection.

☒ Life sciences ☐ Behavioural & social sciences ☐ Ecological, evolutionary & environmental sciences

For a reference copy of the document with all sections, see nature.com/documents/nr-reporting-summary-flat.pdf

Life sciences study design

All studies must disclose on these points even when the disclosure is negative.

Sample size	technical triplicates and biological duplicates or triplicates (as stated) were obtained for all measurements. This represents the accepted norm for a biochemical study, allowing standard deviation and mean to be calculated.
Data exclusions	no data was excluded
Replication	Biological and technical replicates were carried out as indicated in the figure legends.
Randomization	Randomization was not applied to the small biochemical datasets studied, in keeping with established norms for this type of study.
Blinding	Blinding was not applied to the small biochemical datasets studied, in keeping with established norms for this type of study.

Reporting for specific materials, systems and methods

We require information from authors about some types of materials, experimental systems and methods used in many studies. Here, indicate whether each material, system or method listed is relevant to your study. If you are not sure if a list item applies to your research, read the appropriate section before selecting a response.

Materials & experimental systems

n/a	Involved in the study	
☐	☒	Antibodies
☒	☐	Eukaryotic cell lines
☒	☐	Palaeontology and archaeology
☒	☐	Animals and other organisms
☒	☐	Clinical data
☒	☐	Dual use research of concern
☒	☐	Plants

Methods

n/a	Involved in the study	
☒	☐	ChIP-seq
☒	☐	Flow cytometry
☒	☐	MRI-based neuroimaging

Antibodies

Antibodies used	anti-cyclase (Bacillus cereus), anti-PspA (Bacilus subtilis), anti-Rny (Bacillus subtilis), anti-HPr (Bacillus subtilis), IRDye® 800CW Donkey anti-Rabbit IgG Secondary Antibody (LI-COR Biosciences; P/N: 926-32213)
Validation	The specificity of the antibodies was tested in Western blots against the respective purified protein and, if applicable, against cell lysate of Bacillus subtilis.
