# Supplementary material for: Reversible conjugation of a CBASS nucleotide cyclase regulates bacterial immune response to phage infection
Source: Nat Microbiol. 2024 Apr 8;9(6):1579–92. doi: 10.1038/s41564-024-01670-5 (PMC11153139; doi:10.1038/s41564-024-01670-5)

Figure 2

a

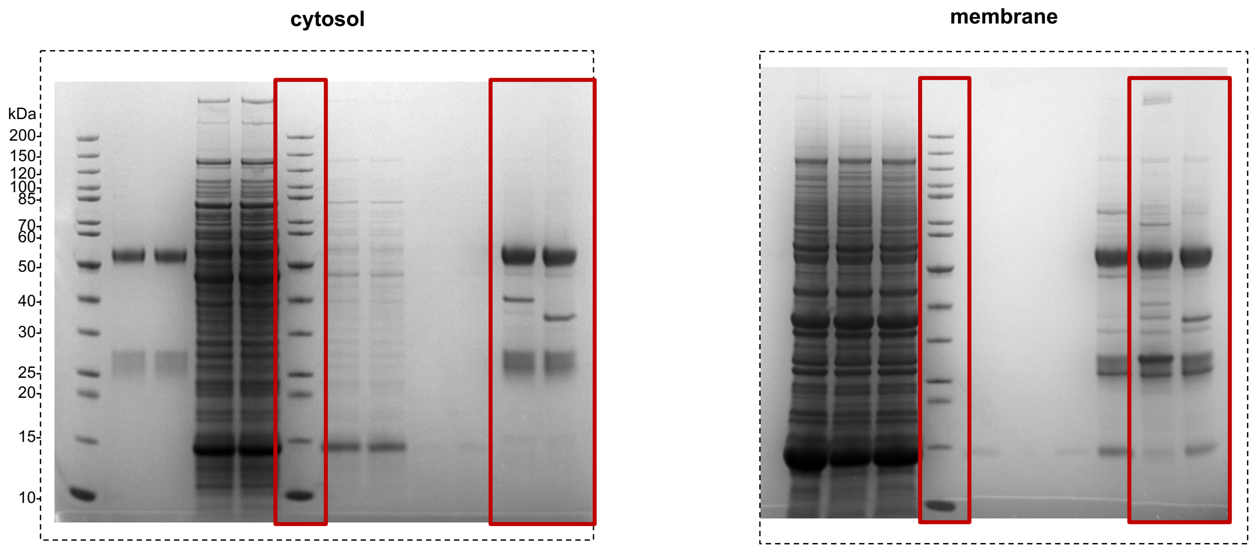
cytosol
membrane
kDa
200
150
120
100
85
70
60
50
40
30
25
20
15
10

d

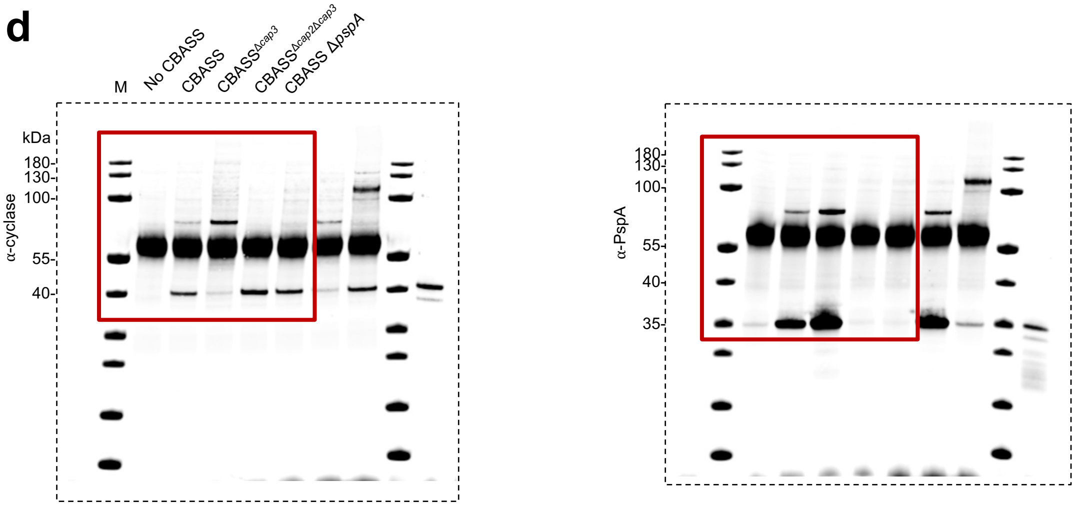
M
No CBASS
CBASS
CBASS$^{\Delta cap3}$
CBASS$^{\Delta cap2 \Delta cap3}$
CBASS Δ*pspA*
kDa
180
130
100
55
40
α-cyclase
α-PspA
35

Figure 3

a

Strep-Cap2		X	X			X	X	
cyclase		X	X	X	X	X	X	X
His-PspA^{Bsu}		X		X		X		X
ATP	M	X	X	X	X			

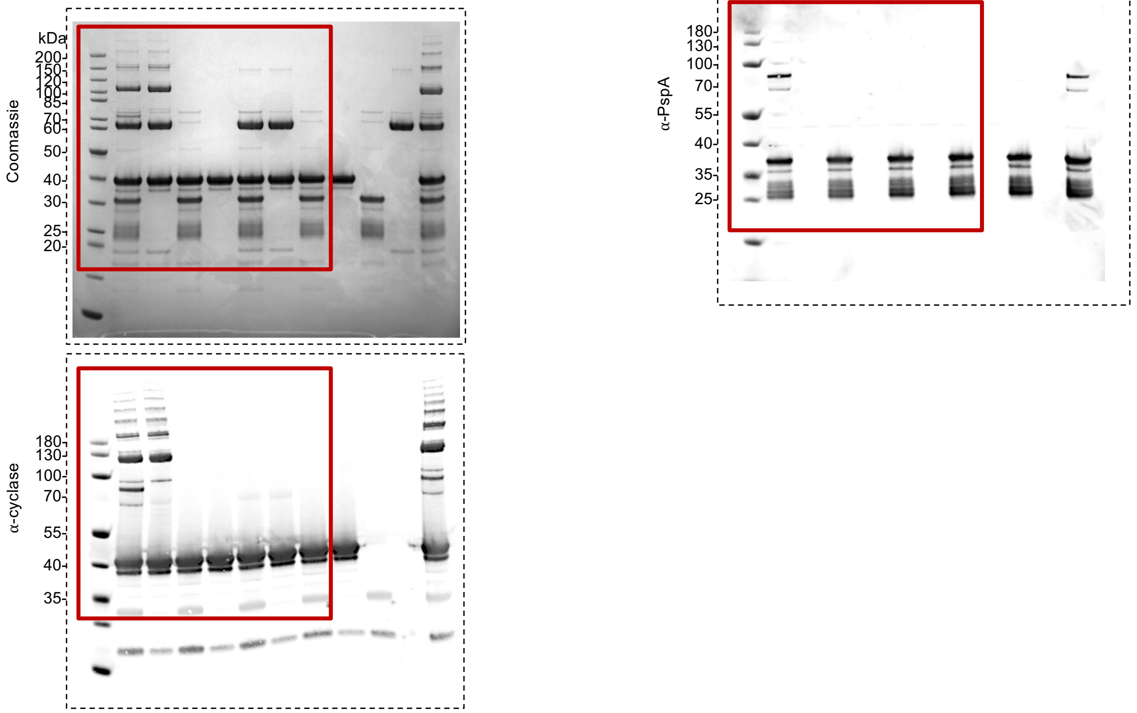

b

Strep-Cap2	M (kDa)	X	X	X	X	X	Cyclase
cyclase		X	X	X	X	X	
PspA^{Bsu}		-	wt	K147R	K220R	K147R K220R	

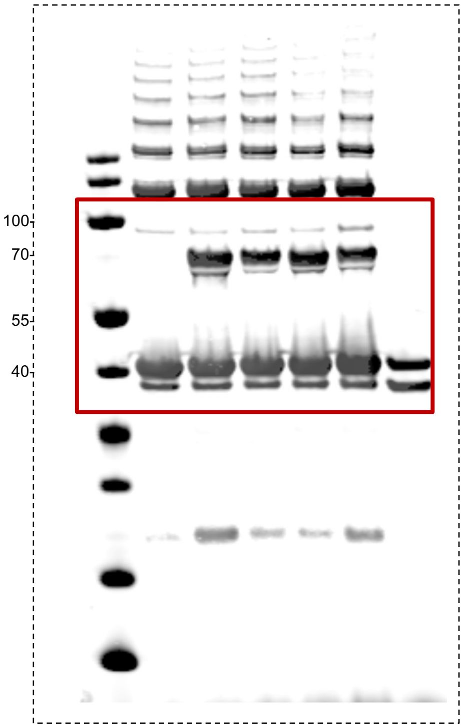

α-cyclase

Figure 4

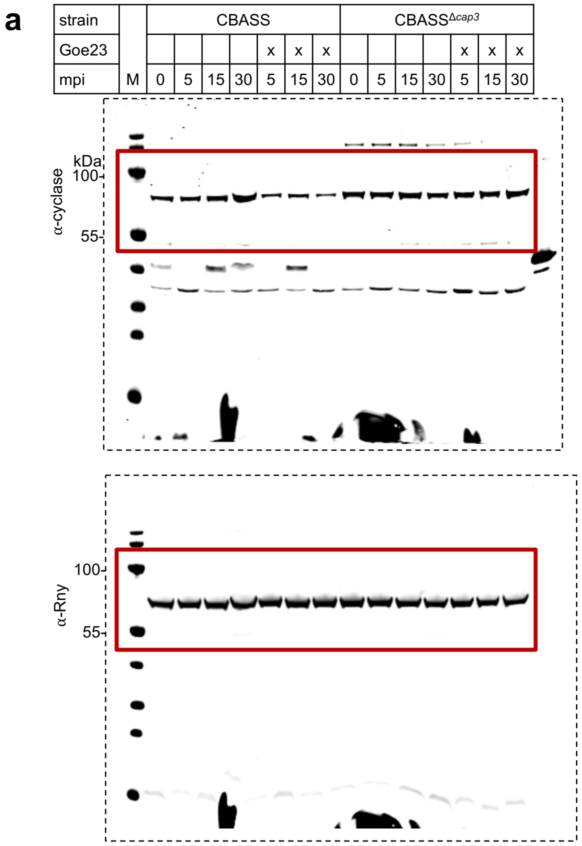
a
strain
CBASS
CBASSΔcap3
Goe23
mpi
M
0
5
15
30
x
kDa
100-
55-
α-cyclase
100-
55-
α-Rny

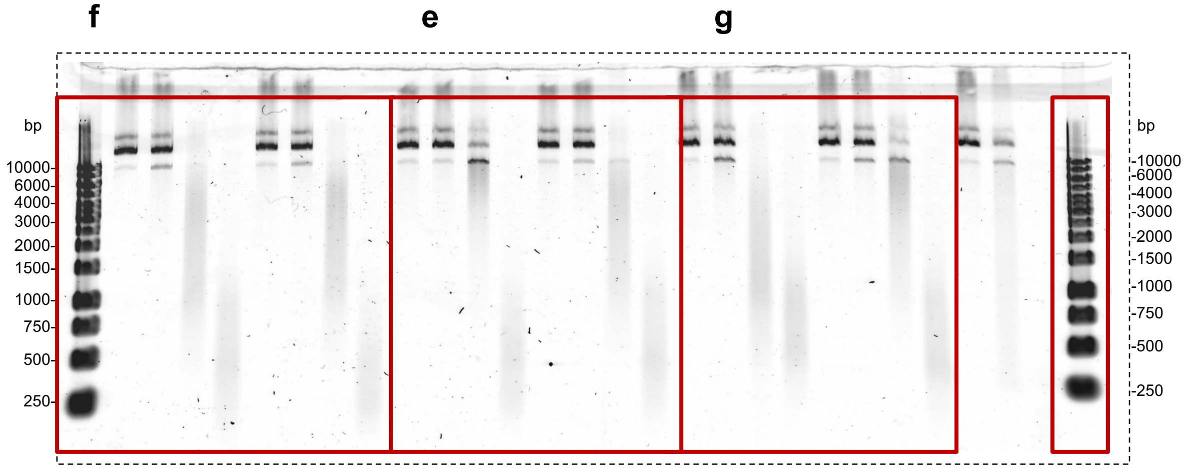
f
e
g
bp
10000-
6000-
4000-
3000-
2000-
1500-
1000-
750-
500-
250-
bp
-10000
-6000
-4000
-3000
-2000
-1500
-1000
-750
-500
-250

Figure 5

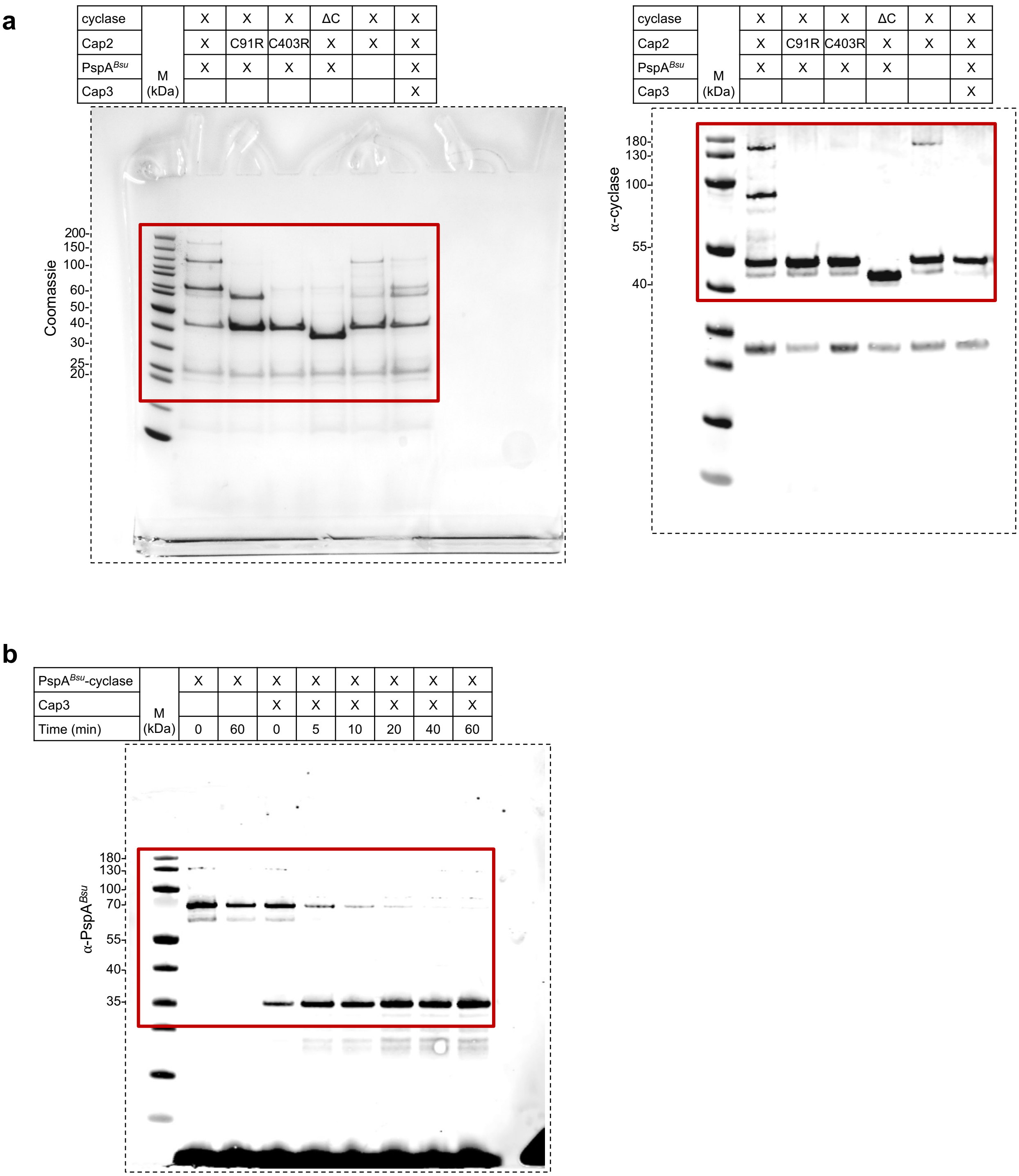
a
cyclase
Cap2
PspA^Bsu
Cap3
M (kDa)
X X X ΔC X X
X C91R C403R X X X
X X X X X
X
Coomassie
200- 150- 100- 60- 50- 40- 30- 25- 20-
α-cyclase
180- 130- 100- 55- 40-
b
PspA^Bsu-cyclase
Cap3
Time (min)
X X X X X X X X
X X X X X X
0 60 0 5 10 20 40 60
α-PspA^Bsu
180- 130- 100- 70- 55- 40- 35-

Extended Data Figure 2

a

	Cytosol						
EV				X	X		
cyclase	X	X				X	X
cyclase$^{\Delta C}$		X		X	X	X	X
fraction	FT		M	W		E	

	Membrane									
EV	X				X			X		
cyclase		X				X			X	
cyclase$^{\Delta C}$			X				X			X
fraction	FT			M	W			E		

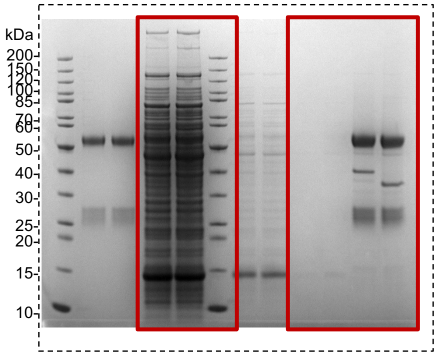

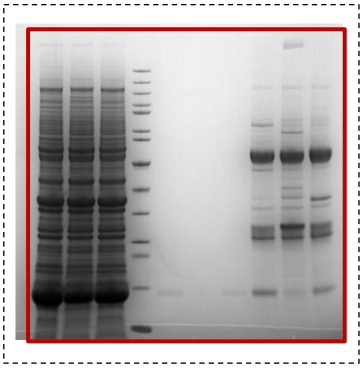

b

	Membrane	
cyclase		X
fraction	M	E

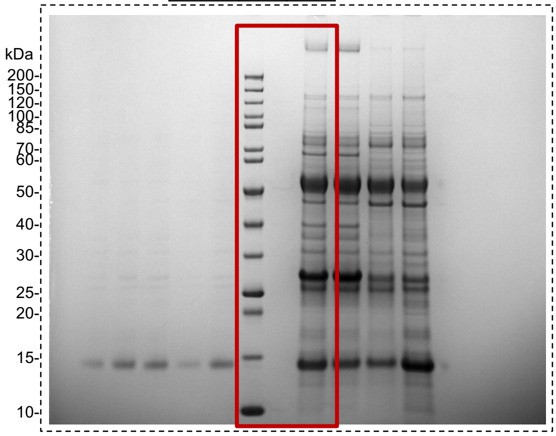

Extended Data Figure 3

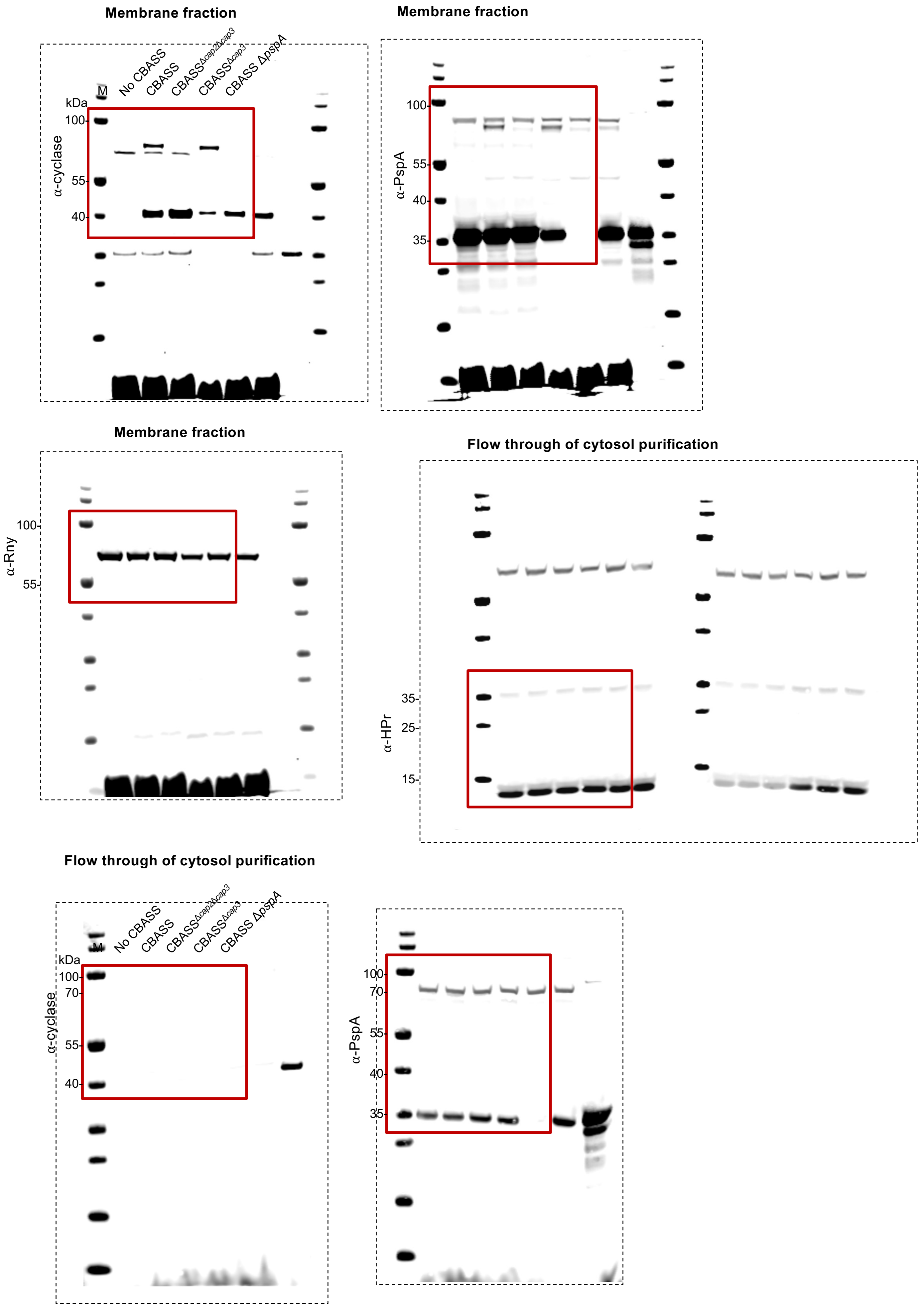

Extended Data Figure 3

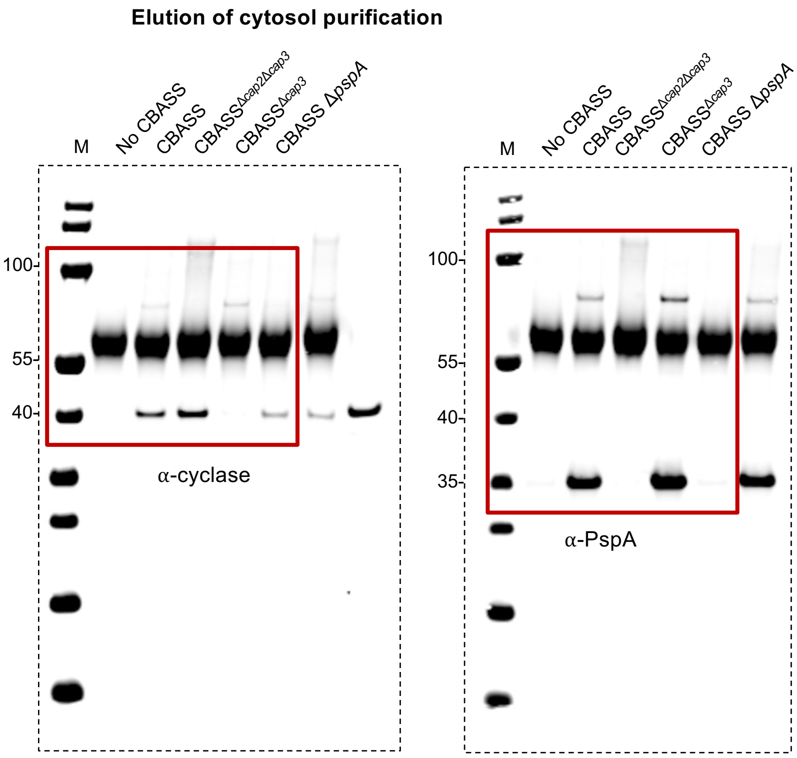

Extended Data Figure 4

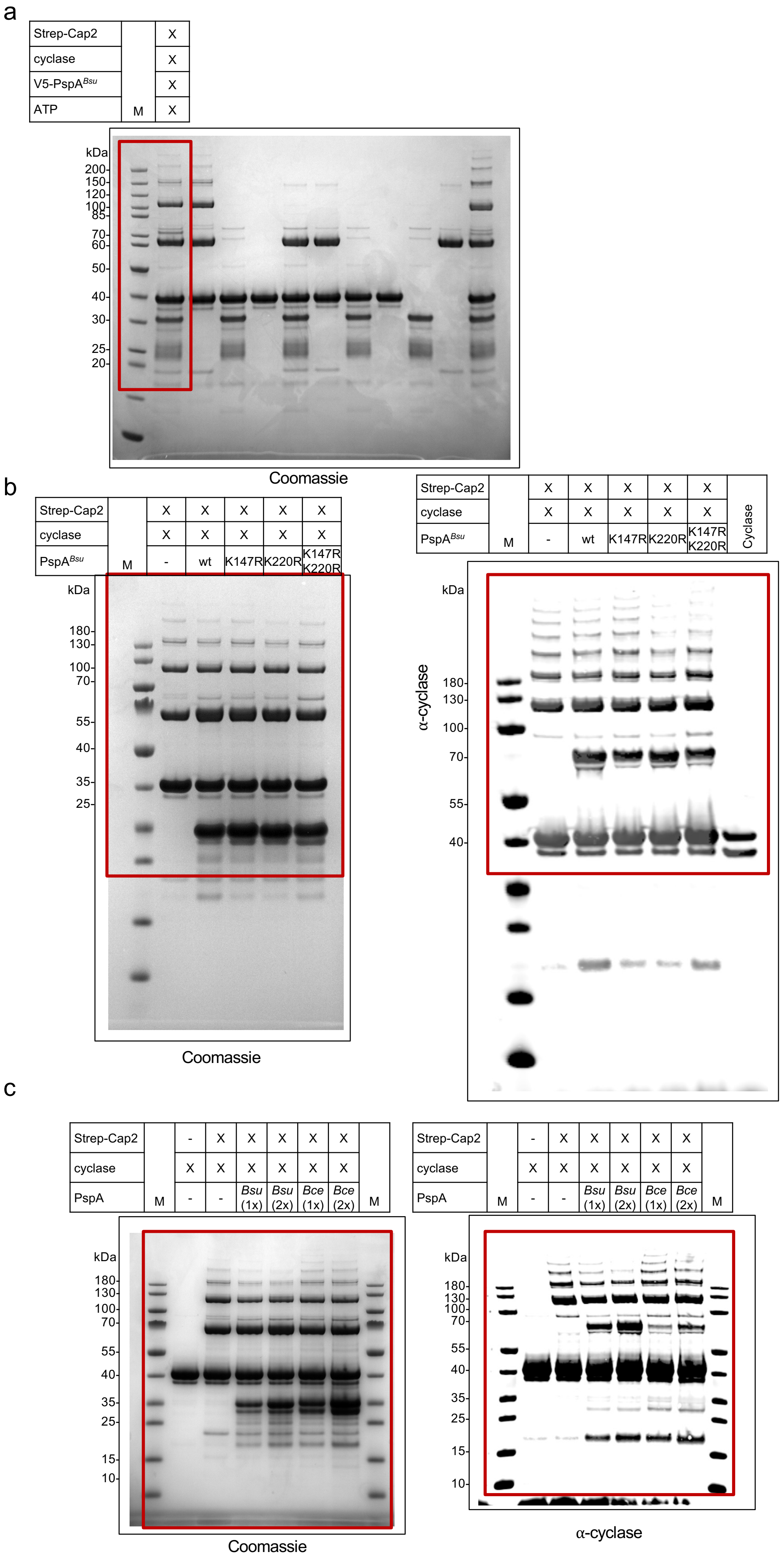

Extended Data Figure 5

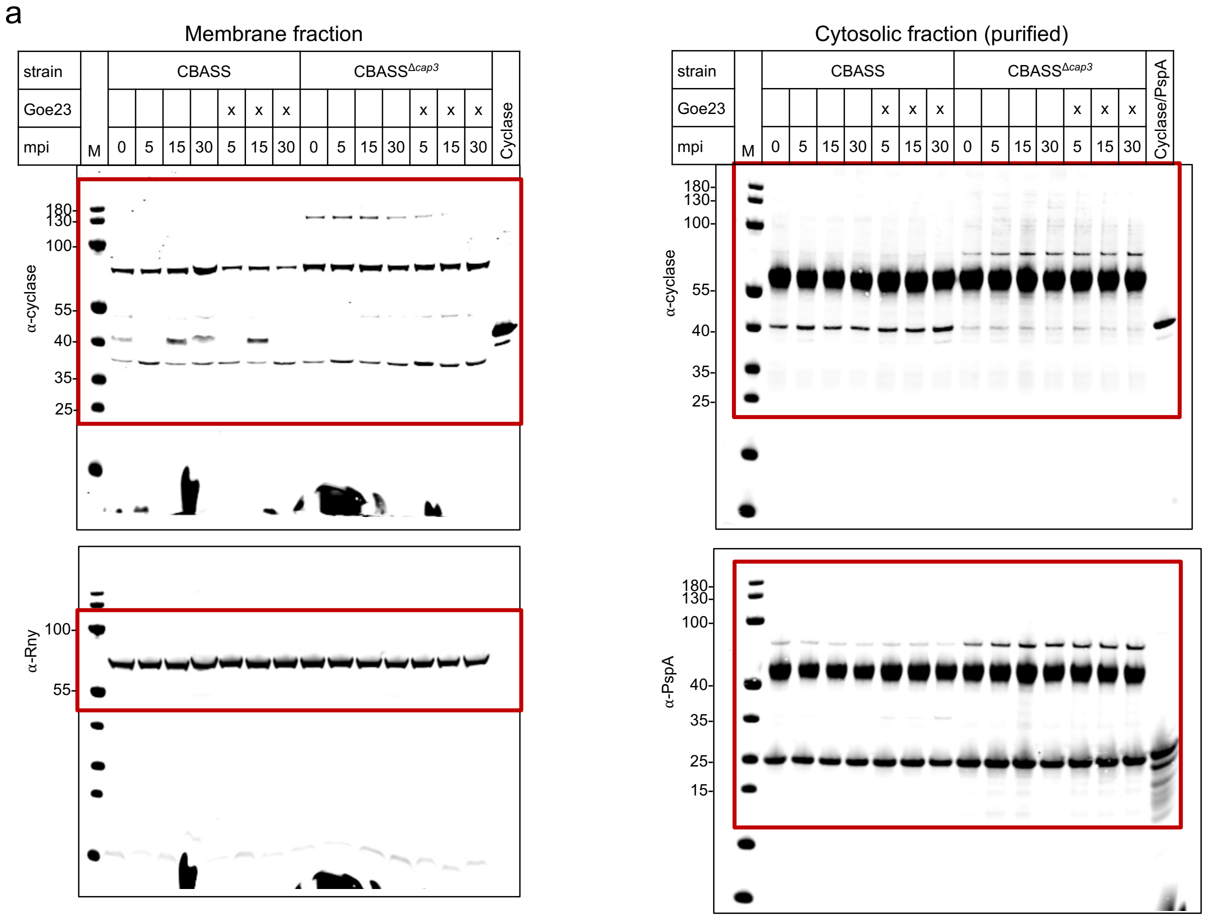

Extended Data Figure 9

a

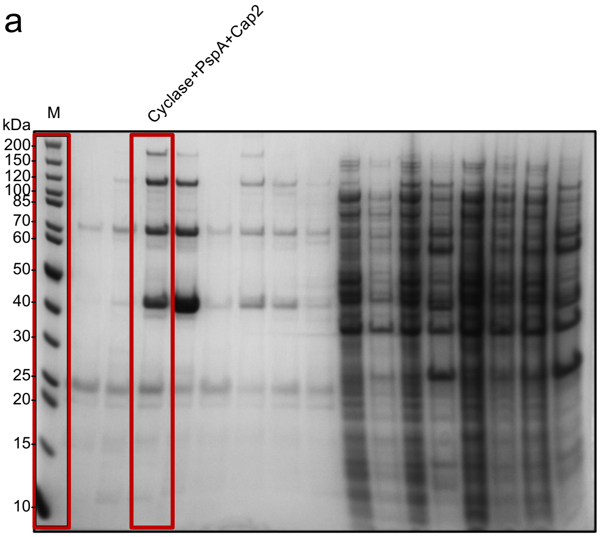

b

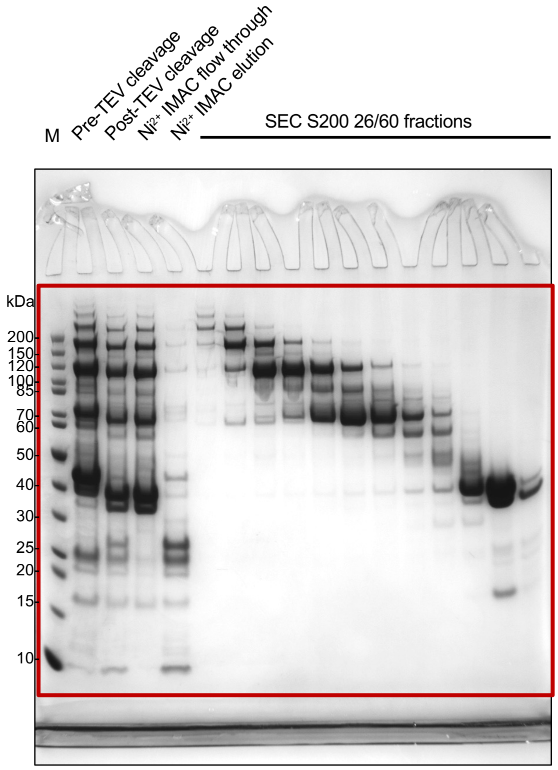

c

PspA-cyclase	M (kDa)	X	X		X	X	X	X	X	X
Cap3				X	X	X	X	X	X	X
Time (min)		0	60	0	0	5	10	20	40	60

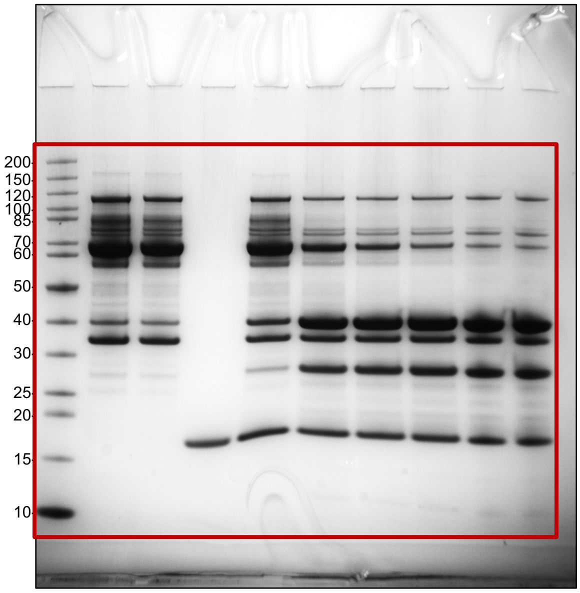

Supplement: Supplementary file 4 — Uncropped gels and blots for all figures. [file 41564_2024_1670_MOESM4_ESM.pdf]
